# Operationalizing intimacy and identity aspects of personality functioning in relation to personality disorder in adolescents

**DOI:** 10.3389/fpsyt.2023.1153274

**Published:** 2023-04-11

**Authors:** Breana R. Cervantes, Sophie Kerr, Salome Vanwoerden, Carla Sharp

**Affiliations:** ^1^Department of Psychology, University of Houston, Houston, TX, United States; ^2^Department of Psychiatry, University of Pittsburgh, Pittsburgh, PA, United States

**Keywords:** parental closeness, intimacy, identity diffusion, AMPD criterion A, self-other understanding, adolescents, borderline personality disorder, ICD-11

## Abstract

According to dimensional models of personality pathology, deficits in interpersonal (intimacy and empathy) and self (identity and self-direction) function (Criterion A) are core to all personality disorders. These aspects of personality functioning (Criterion A) have seldom been evaluated for how they might relate to one another in the context of personality pathology in adolescents. Moreover, the use of performance-based measures to evaluate aspects of Criterion A function remains an untapped resource. Therefore, the present study aimed to evaluate relations between two features of Criterion A, maladaptive intimacy and maladaptive (or diffused) identity, in adolescence. For intimacy, we leverage a performance-based approach to studying intimacy, operationalized in a developmentally relevant way (perceived parental closeness). For identity, we rely on a validated self-report measure of identity diffusion. We examined the relationship between these features with each other and their relations with borderline features. Additionally, we explored whether identity diffusion mediated the expected relationship between perceived parental closeness and borderline features. We hypothesized that greater distance in perceived parental closeness would be associated with higher levels of borderline features, as well as higher levels of identity diffusion, and that identity diffusion would account for the relationship between intimacy and personality pathology. The sample included 131 inpatient adolescents (*M*_age_ = 15.35, 70.2% female). Results indicated that intimacy, operationalized as perceived parental closeness, with both mothers and fathers was significantly associated with levels of identity diffusion and borderline features. In addition, greater feelings of closeness with parents were associated with lower severity of borderline features *via* healthier identity function. Implications of the results, limitations, and future directions are discussed.

## Introduction

Research has demonstrated that borderline personality disorder (BPD) in adolescence has notable similarities to adult BPD, in terms of impairments in functioning, psychiatric comorbidities, phenomenology, and prevalence (1–4). Compared to other adolescent mental disorders, adolescent BPD is associated with higher rates of self-harming behaviors, suicidality, and impulsivity, highlighting the severity of this disorder (5, 6). Extensive literature has also documented the adverse long-term outcomes for adults with BPD, including premature mortality, poor quality of life, and poor social and occupational functioning. Additionally, there is mounting evidence of poor psychological, social, vocational, and physical health outcomes for adolescents and emerging adults with BPD and ‘subthreshold’ BPD [e.g., (7–10)]. Consequently, there is a growing emphasis on early detection and intervention of BPD in adolescence (11).

While the BPD construct has been helpful to legitimize the early diagnosis and treatment of BPD in young people (12), the personality disorder field has been moving toward a dimensionalized diagnostic system as represented by the 11th version of the International Classification of Diseases (ICD-11) and the Alternative Model for Personality Disorders (AMPD) in Section III of the *Diagnostic and Statistical Manual of Mental Disorders, Fifth Edition* (13, 14). Both the ICD-11 and AMPD are thought to have significant developmental relevance due to their focus on self and interpersonal functioning—two key processes associated with the adolescent developmental period. Specifically, according to the AMPD, the core and common feature of all personality dysfunction is defined by Criterion A, which describes impairments in self-functioning (identity and self-direction) and interpersonal functioning (empathy and intimacy). This focus is also reflected in the ICD-11 entry criterion. For the assessment of AMPD Criterion A, a range of self-report and interview-based measures have been developed. These measures show strong validity and reliability in adults [see (15) and (16) for a review] and there is an emerging literature validating Criterion A-related measures in adolescents [e.g., (17–20)]. The limitations of self-report measures to adequately assess Criterion A function, given the known impairment in self-reflection associated with personality pathology, have been noted (21, 22), and the potential of using experimental or more performance-based measures has been suggested—in particular for Criterion A (23). Such measures are thought to be less subject to a person’s self-presentational efforts (24). In addition, because individuals construct their responses to the task, in lieu of selecting descriptions on a Likert scale that best fit them, performance-based measures are thought to provide a more idiographic picture of personality (25). Bornstein (21) also notes that many aspects of personality functioning are hard to verbalize. This may be especially true for adolescents who, by virtue of their developmental phase, may be struggling to express themselves (26). In the context of adolescent personality pathology, the question then becomes how to operationalize and assess aspects of Criterion A in a developmentally relevant way through measures that do not rely so heavily on verbalization capacity.

In the current study, we approach two aspects of Criterion A functioning—identity and intimacy—in the following way. For identity assessment we rely on a validated self-report instrument of identity diffusion in adolescents. For intimacy, we considered the fact that for adolescents, a developmentally salient relationship is that of the parent-adolescent relationship (27). While adolescents begin to gravitate toward peer relationships, research has shown the parent-adolescent relationship to still be the most relevant attachment context for adolescents (28, 29). Indeed, in the context of adolescent personality pathology, Skabeikyte-Norkiene and colleagues (30) confirmed that relationship quality (closeness and discord) with parents but not peers accounted for the variance in impaired levels of personality functioning in adolescents. This underscores the continued importance of the parent–child relationship, in particular, as a protective factor for buffering against the risk for psychopathology in adolescence, as young people navigate the additional stressors (i.e., neurobiological, social, and emotional changes) brought on by the transition to this developmental phase (31–33).

Beyond operationalizing intimacy in adolescence in a developmentally salient manner through a focus on the parent-adolescent relationship, we also sought to assess this construct with a performance-based approach used to assess typical intimacy functioning. We do this to evaluate the usefulness of performance-based measures to assess aspects of Criterion A (e.g., maladaptive identity) in adolescents. A well-used measure in social psychology for assessing intimacy is the Inclusion of Others in Self (IOS) Scale, a pictorial scale capturing the subjective sense of interconnectedness between the self and other through a series of progressively more overlapping Venn-like diagram circles, ranging from no overlap to full overlap in circles (34). Greater overlap, or less distance between the centers of the two circles, suggests greater feelings of perceived closeness to the other. In adolescent studies, the IOS has often been employed as an instrument for operationalizing relational closeness to parents, peers, and community in a multitude of contexts, without relying on verbalization. Indeed, several empirical studies have evaluated adolescents’ self-parent overlap in the context of psychopathological (e.g., depression, sexual risk-taking), emotional (e.g., self-esteem, life satisfaction, positive and negative emotionality), and neural (e.g., ventral striatum activity) outcomes (35–39). As yet, no studies have used the IOS to operationalize intimacy (perceived parental closeness) in relation to adolescent personality pathology. However, researchers have utilized the IOS in studies of adults with BPD as a measure of connectedness with romantic partners following discussions of threatening topics (40), theoretical others following exposure to social inclusion, “over-inclusion,” and ostracism conditions (41), and theoretical others following participation in multisensory perceptual self-other-distinction tasks (i.e., facial morphing task and synchronous or asynchronous interpersonal multisensory stimulation) (42).

Against this background, our first aim was to evaluate the relationship between maladaptive intimacy in adolescents, operationalized through IOS measurement of perceived parental closeness, and more traditionally measured personality pathology—in this case borderline features. Demonstrating this relationship would confirm that intimacy, operationalized thus, relates to personality pathology in adolescents and can be used to evaluate a core feature of personality functioning in adolescents. We expected greater distance in perceived closeness to both mothers and fathers (i.e., less feelings of parental closeness) to be associated with higher levels of borderline features. Indeed, while no studies have evaluated subjective parental closeness in relation to adolescent borderline features or broader personality dysfunction by way of the IOS scale, links between lower levels of parent-adolescent closeness and greater impairment in Criterion A level of personality functioning have been established (30). Moreover, prior research has shown similar associations between negative interactions with parents, such as less parental closeness, parental warmth, perceptions of parents as caring, maternal emotional support and greater boundary violations, and adolescent personality dysfunction [e.g., (43–47)].

Our second aim was to investigate the association between intimacy (perceived parental closeness) and identity diffusion. While Criterion A is intended to be viewed as a unidimensional severity criterion (15), from a more psychodynamic, process-oriented view of personality, we would expect that self and interpersonal functioning would be in constant, reciprocal interaction with one another. In fact, developmental theories of BPD, specifically mentalization-based theory (48), suggest that it is through high quality interactions with the caregiver in which a child is mentalized, that a sense of self begins to emerge. Through constant feedback, recognition and description from caregivers commenting and enquiring about the motivations and intentions linked to children’s behavior, children begin to form a representation of self. This process culminates in adolescence, a developmental phase where identity formation becomes crucial to resolve a crisis between identity diffusion (“a lack of integration of the concept of self and significant others”) and integration (consolidation of one’s representations of the self across time and contexts) (49, 50). As articulated in the AMPD, identity diffusion involves an absence of agency or experience of the self as unique, inappropriate boundaries with others, weak or threatened self-esteem and distortions in self-appraisal, and emotions incongruent with the internal experience or context (14). In contrast, identity consolidation involves an ongoing awareness of the self, the ability to maintain role-appropriate boundaries, consistent and regulated self-esteem and accurate self-appraisal, and the ability to experience, tolerate, and regulate a full range of emotions (14). Moreover, previous research has demonstrated associations between parent-adolescent relationships, including perceptions of parental care vs. overprotection and family cohesion, and adolescent identity status (diffusion, foreclosure, moratorium, achievement). Specifically, links between greater perception of paternal care and lower identity diffusion, greater perception of paternal and maternal care and higher foreclosure, and greater family cohesion and lower diffusion and moratorium have been shown among adolescents and young adults (51, 52). Thus, adolescence is a vital period for both identity formation and optimal parent–child relations. Against this background, we expected a significant association between intimacy (perceived parental closeness) and identity diffusion so that greater distance in perceived parental closeness (i.e., less feelings of parental closeness) would be associated with higher levels of identity diffusion. We also expected identity diffusion to relate to borderline features—a finding which has already been established in adolescence (53–55).

Finally, we were interested in investigating whether identity diffusion explains some of the variance in the expected relationship between intimacy and personality pathology. Following Fonagy et al.’s (48) model described above and principles of attachment theory, one could argue that perceived parental closeness should relate to identity diffusion in adolescents. On this basis, we expected that identity diffusion would account for significant variance in the hypothesized relationship between lack of closeness with parents and increased borderline features.

## Materials and methods

### Participants and procedure

The sample was drawn from a larger study on the social-cognitive correlates of psychopathology. Participants were recruited from an inpatient psychiatric unit at a private hospital in a major city in the southwestern United States. Inclusion criteria required fluency in English to consent and complete the assessments and being between 12 and 17 years old. Exclusion criteria were active psychosis or mania, an autism spectrum disorder, or an IQ of less than 70. *N* = 131 adolescents (70.2% female, *M_age_* = 15.35, *SD* = 1.43) on the unit underwent the IOS assessment alongside measures of identity diffusion and personality pathology. The sample had the following racial/ethnic breakdown: 67.2% White/not Hispanic (*n* = 88), 5.3% Hispanic (*n* = 7), 5.3% Asian (*n* = 7), 1.5% African American (*n* = 2), 3.8% mixed or other (*n* = 5), and 16.8% unspecified (*n* = 22). Average length of stay was 36.94 days (*SD* = 13.20). The study was approved by the institutional human subjects review committee. Adolescents and their parents provided assent and consent, respectively. Adolescents were assessed by doctoral-level clinical psychology students and/or trained clinical research assistants during the first 2 weeks following admission. The study was not preregistered as data collection took place in 2015 when less emphasis was placed on open science practices.

### Measures

#### Borderline personality disorder features scale for children

The Borderline Personality Disorder Features Scale for Children (BPFS-C) is a 24-item self-report measure of borderline features in children and adolescents (56). Items are rated on a 5-point Likert-type scale, ranging from 1 (*not true at all*) to 5 (*always true*) to assess how participants feel about themselves and others. The total score is calculated by summing all items, with higher scores indicating higher levels of BPD features. The scale also yields four subscales: identity problems (“I feel that there is something important missing about me, but I do not know what it is”), affective instability (“I go back and forth between different feelings, like being mad or sad or happy”), negative relationships (“I worry that people I care about will leave and not come back”), and self-harm (“I get into trouble because I do things without thinking”). We use the total score, which has demonstrated reliability and validity in previous studies with adolescents [e.g., (57, 58)]. Cronbach’s alpha for the total score in the current sample was α = 0.89.

#### Inclusion of other in the self

The original Inclusion of Other in the Self (IOS) was a single-item pictorial measure designed to assess how individuals conceptualized their experience of relational closeness to a target (34). The original scale depicted seven pairs of circles (one circle designated the “self” and the other designated the “other”), with the sets ranging from completely separate to nearly completely overlapping and individuals selecting the pair that best represents their relationship. Researchers may choose to score responses on a 7-point scale, with increasingly overlapping circles corresponding to higher scores to suggest greater feelings of perceived closeness to the “other.” Previous studies with adults report high levels of reliability and validity in terms of relations with other measures of relationship closeness (34, 59, 60), including test–retest reliability and convergent validity with the Relationship Closeness Inventory (61) and the Subjective Closeness Index (62). The IOS has also been used with samples of preschool-aged children, school-aged children, and adolescents (63–68). Importantly, due to the IOS’s flexibility in assessing both relationships (e.g., peers, romantic partners, parents, God) and broader contexts (e.g., culture, nature, consumer brands); its minimal reliance on language; and its ability to sidestep biases elicited by verbal self-report measures, it is ideal for developmental downward extension to youth (34, 69). The current study used the Continuous IOS (70), an online version of the IOS where participants drag one circle labeled the “self” toward or away from a circle (designated as mother or father) to best represent their relationship. Each participant completed the IOS twice, once in reference to their mother and once in reference to their father. The Continuous IOS grants the participant finer precision in indicating their experienced degree of relational distance in that it yields an output for percentage overlap (from 0% to 100%) between the two circles, as well as an output for distance between the two circles, ranging between −100 and 100, where −100 represents circles as far apart as possible, 0 represents adjacent circles, and 100 represents circles nearly completely overlapping. The “distance between centers” output is considered particularly valuable, as it allows for clearer distinction between participants “who represent their relationship as two merely tangent circles” vs. those “who see their relationship as two wholly separate circles” (69). In the present study, distance scores were obtained and made positive by adding 100 to each to aid in interpretability. Thus, higher distance scores indicate greater distance in perceived parental closeness (i.e., less feelings of perceived parental closeness). Importantly, although higher scores could perhaps be interpreted as an inflated sense of closeness that is driven by impairments in self-other distinction, a key feature of BPD, previous studies employing the IOS with individuals with BPD have demonstrated that BPD patients reported less feelings of closeness than their healthy control counterparts. Specifically, individuals with BPD perceived less social connection to theoretical others irrespective of being in a social inclusion, “over-inclusion,” or ostracism condition (41), and a sharper decline in feelings of closeness to romantic partners when they were observed to demonstrate a heightened stress response (40).

### The assessment of identity development in adolescents

The Assessment of Identity Development in Adolescents (AIDA) is a 62-item self-report questionnaire of identity development in adolescents that is specifically focused on impairments in personality functioning (71). Items are rated on a 5-point Likert scale and yield a total score representing identity diffusion and two scale scores representing identity discontinuity (“I can imagine the kind of person I will be in the future,” “I feel I do not really belong anywhere”) and identity incoherence (“I often feel lost, as if I had no clear inner self,” “I need reassurance from others to not give up”). The current study uses the total score (identity diffusion). The total score has shown evidence of reliability and validity in variety of samples across cultural groups, including a large US community sample and a clinical subsample of the larger study from which the current sample was drawn (19, 72, 73). In the current study, of the 131 total study participants, *n =* 109 completed this measure. McDonald’s omega indicated good internal consistency (ω = 0.88).

### Data analytic strategy

All analyses were conducted using SPSS 25 (74). Prior to main analyses, descriptive statistics were examined to evaluate assumptions of normality. Next, attrition analyses were calculated to examine whether there were systematic differences in age, gender, perceived closeness to mothers (IOS—mother), perceived closeness to fathers (IOS—father), and borderline features (BPFS-C) between adolescents that completed the measure of identity diffusion (AIDA) and those that did not. Bivariate correlations were tested between main study variables. Finally, we tested a mediation model using the PROCESS macro with borderline features as the dependent variable and gender as a covariate. Two separate models were tested with closeness to either mother and father as independent variable and identity diffusion as the mediator. Bias-corrected and accelerated 95% confidence intervals based on 1,000 bootstrapped samples were examined to evaluate the indirect effect of parental closeness on borderline features *via* identity diffusion.

## Results

### Aims 1 and 2: Associations between intimacy, identity diffusion, and borderline traits

For continuous variables (age, IOS—mother, IOS—father, BPFS-C), independent sample *t*-tests were conducted between AIDA completers and non-completers, and for the categorical variable (gender), a Chi-square test was conducted. Results showed no significant differences between AIDA completers and non-completers for age [*t*(129) = −0.61, *p* = 0.543], IOS—mother [*t*(129) = −0.213, *p* = 0.832], IOS—father [*t*(128) = −1.11, *p* = 0.268], or BPFS-C [*t*(124) = 0.75, *p* = 0.453]. However, significant differences between AIDA completers and non-completers were found for gender, [χ^2^(1, *N* = 131) = 10.871, *p* < 0.001], and therefore gender was controlled for in subsequent mediation analyses.

Table 1 displays bivariate correlations between main study variables as well as with demographic variables of gender and age. Gender and age were not significantly related to any of the main study variables but were associated with each another. Reports of closeness with mothers and fathers were highly related.

**Table 1:** Bivariate correlations between main study variables.

	1	2	3	4	5	6
1. Closeness—mom	-					
2. Closeness—dad	0.56^**^	-				
3. Identity diffusion	−0.23^*^	−0.32^**^	-			
4. BPD features	−0.18^*^	−0.25^**^	0.73^**^	-		
5. Age	−0.07	−0.11	0.09	0.17	-	
6. Gender (female)	0.02	0.004	−0.10	−0.14	0.25^**^	-
Mean (SD)	138.88 (45.27)	129.58 (45.01)	110.13 (41.45)	68.53 (14.66)	15.35 (1.43)	-
% Female	-	-	-	-	-	72.2
*N*	131	130	109	126	131	131

^*^p ≤ 0.05, ^**^p < 0.01.

Regarding our first hypothesis—that intimacy would be related to personality pathology, our results showed a significant negative correlation between perceived closeness with father and borderline traits (moderate effect size), as well as perceived closeness with mother and borderline traits (small effect size).

Regarding our second hypothesis—that intimacy would be related to identity diffusion, results showed significant negative correlations (moderate effect size) between perceived parental closeness and identity diffusion for both mothers and fathers. Identity diffusion was also found to be strongly related to borderline features (large effect size).

### Aim 3: The role of identity diffusion in explaining variance in the relationship between intimacy and personality pathology

For both models testing closeness to mothers and fathers, parameter estimates are displayed in Table 2.

**Table 2:** Mediation analyses.

Path	*B*	*SE*	*t*	*P*	LLCI	ULCI
IV: Closeness—Mother^a^						
a	−0.22	0.09	−2.49	0.014^*^	−0.39	−0.04
b	0.26	0.02	10.64	0.000^**^	0.21	0.31
c	0.00	0.02	0.07	0.943	−0.04	0.05
c’	−0.06	0.03			−0.11	−0.01
IV: Closeness—Father^b^						
a	−0.31	0.09	−3.45	0.001^**^	−0.48	−0.13
b	0.26	0.03	10.28	0.000^**^	0.21	0.31
c	−0.02	0.02	−0.66	0.513	−0.06	0.03
c’	−0.08	0.02			−0.13	−0.03

^a^*n* = 108; ^b^*n* = 107. Values are unstandardized path coefficients from models including gender as a covariate. ^*^p < 0.05, ^**^p < 0.01.

In the first model, perceived parental closeness to mothers (IOS—mother) was entered as the independent variable, borderline features (BPFS-C) served as the dependent variable, and identity diffusion (AIDA) was explored as a mediator. Gender was entered as a covariate. Naturally mirroring the bivariate analyses, the mediation analysis revealed that perceived parental closeness to mothers was significantly negatively associated with levels of identity diffusion, and identity diffusion was significantly positively associated with severity of borderline features. Additionally, gender was not associated with levels of identity diffusion or borderline features. While there was no direct relation between closeness with mothers and severity of borderline features, the indirect path was statistically significant, such that reduced identity diffusion explained significant variance in the relationship between perceived parental closeness to mothers and borderline features. Put differently, greater perceived closeness with mothers was significantly associated with lower levels of identity diffusion, which, in turn, was related to less borderline features.

In the second model, parental closeness to fathers (IOS—father) served as the independent variable, borderline features (BPFS-C) served as the dependent variable, identity diffusion (AIDA) was entered as the mediator, and gender was entered as a covariate. Again, mirroring the bivariate analyses, results indicated a significant negative association between perceived parental closeness to fathers and levels of identity diffusion, and a significant positive association between identity diffusion and severity of borderline features. Gender was not associated with levels of identity diffusion or borderline features. Moreover, the indirect path was statistically significant, with confidence intervals that did not include zero. Thus, greater perceived closeness with fathers was significantly associated with less borderline features *via* lower levels of identity diffusion. In other words, reduced identity diffusion explained significant variance in the relationship between perceived closeness to fathers and borderline features *via* less identity diffusion.

## Discussion

The present study aimed to examine relations between two aspects of AMPD Criterion A functioning, intimacy and identity, and borderline features in a sample of inpatient adolescents by utilizing a performance-based measure of intimacy (relational closeness) and a self-report measure of identity diffusion. To this end, we used the IOS to operationalize intimacy specifically in the context of parent-adolescent relationships (i.e., assessing perceived parental closeness as a proxy for intimacy), given the still emerging relevance of intimacy with romantic partners in adolescence. Thus, we were interested in evaluating associations between perceived parental closeness and identity diffusion (Aim 1), perceived parental closeness and borderline features (Aim 2), and identity diffusion as a mediator in the association between perceived parental closeness and borderline features (Aim 3). We expected our findings to also have relevance for ICD-11 personality disorder application of criteria, given the conceptual overlap between the entry criteria of ICD-11 and AMPD.

Our results revealed that intimacy, operationalized as perceived closeness with both mothers and fathers, related to borderline traits, such that greater parental closeness was associated with lower BPD severity, thereby confirming its relevance for personality pathology in adolescence. This finding is comparable with previous research linking lower levels of parental closeness and greater impairment in level of personality functioning among adolescents (30). Our results also confirmed a relationship between intimacy and identity diffusion, suggesting that adolescents who reported feeling closer to their parents were more likely to report lower levels of identity diffusion. In addition, our findings confirmed a relationship between identity diffusion and borderline features, which is not unexpected given that identity disturbance is a core feature of BPD and similar findings have been demonstrated by prior research (19, 50, 73, 75, 76). Moreover, intimacy exerted a statistically significant indirect effect on borderline features *via* identity diffusion, such that greater perceived parental closeness was associated with lower BPD severity *via* lower levels of identity diffusion, but with no direct effect on borderline features in the absence of identity diffusion.

From a methodological standpoint, our results support assertions that emphasize the complementary value of performance-based measures [e.g., (21–23)], especially those that do not rely on verbal capacity (21), for the assessment of personality functioning in adolescents. Bornstein (21) has written extensively about the value of performance-based or more process-oriented measures of personality pathology. We agree with his arguments especially with regard to Criterion A, which was intended by its architects to represent the more psychodynamic, process-oriented aspect of personality functioning (77). Bornstein argues that self-report alone may inflate co-occurrence with other disorders given the tendency of individuals to follow a particular pattern in answering questions across domains that actually represent different domains of functioning. Personality assessment that also includes performance-based measures may therefore add incremental value to assessment through self-report. Further elaborating the process-oriented aspects of personality functioning, Weiner (22) has pointed out that performance-based measures, experimental tasks, as well as interview-based measures, require face-to-face interaction with individuals being evaluated. We have argued that personality lives in the intersubjective space between people (78), which means that to assess it fully, one has to be in interaction with another human being to show itself. Although the IOS in the current measure did not require actual interaction, its value lies in that it begins to approximate relationship functioning perhaps in a more ecologically valid way compared to self-report.

Our findings also provide some support for the hypothesized link between self and interpersonal aspects of Criterion A, albeit preliminary given the cross-sectional nature of our data (see limitations below). Several theories of personality functioning promote the idea that self and interpersonal functioning are inextricably linked (79–81). Guided by Fonagy et al.’s (48) developmental model for the development of BPD, we have postulated that the caregiving environment influences self and identity development, which in turn determines healthy and/or unhealthy personality functioning. That identity diffusion explained a significant amount of variance in the relationship between perceived parental closeness and borderline features provides some support for this idea.

The limitations of this study should be taken into account. First, a central limitation is that the interpretation of the study’s findings is constrained by its cross-sectional design, and therefore, conclusions about causation cannot be made. Second, while the IOS is considered a performance-based measure, it still has quite a bit of face validity. Therefore, the IOS is a performance-based tool to the extent that it does not rely on verbalization, and it asks an individual to “do” something. However, the IOS certainly still has some face validity in the sense that individuals may understand what they are revealing about themselves when they complete it. Other performance-based measures such as projective testing, in contrast, are thought to reveal those dynamics not consciously accessible to individuals and should be examined further for their value in assessing Criterion A components in adolescents. Third, the generalizability of our findings is limited by the fact that this sample was drawn from a private, inpatient psychiatric unit for adolescents. Therefore, future studies considering building upon our work can address these limitations by testing this mediational model in various demographically diverse, clinical and community samples, particularly within a longitudinal design. Researchers may also consider incorporating additional measures of psychopathology as well as other developmentally relevant variables (e.g., parent–child attachment, peer attachment, IOS-closeness with peers) utilizing self-report, interview-based, and experimental or task-based methods of assessment; in so doing, assessing the incremental value of IOS-based parental closeness alongside other measures of parental, peer, or romantic intimacy. Further, while we contend that relationships with parents are still the most intimate compared to other relationships in adolescence, we also note that adolescence marks a transitional period in which the relationships outside the home become more intimate and stable. Thus, future considerations should be made regarding whether to conceptualize parent-adolescent closeness as a feature of Criterion A or intimacy, which is defined as the capacity for mutually rewarding relationships. Relatedly, the present study’s theoretical lens was based in attachment theory, which informed our position to investigate identity diffusion as a mediator of the relationship between parental closeness and borderline traits. However, alternative theoretical positions (e.g., developmental psychology) might suggest different study hypotheses and should therefore be explored in future research. Finally, it is noteworthy that while research utilizing the IOS in the context of BPD seems to suggest that the IOS taps into a measure of relationship closeness where healthy functioning is reflected by close circles and unhealthy relationships are indicated by distant circles, other measures of relationship function may instead show “enmeshed” relationships associated with personality pathology. Consequently, researchers should consider this impairment in self-other distinction that is characteristic of personality pathology when operationalizing intimacy.

Despite these limitations, the current study’s findings uniquely contribute to the literature base on assessment of Criterion A constructs in adolescents. Moreover, the use of the IOS to assess parental closeness is especially salient within developmental psychopathology research as it hones in onto a particular type of intimacy (parental closeness) that begins to change throughout adolescence as new peer and romantic relationships take the forefront, despite remaining critical for scaffolding optimal psychological and social-cognitive development. We contend that the IOS provides an efficient (it takes 1 min), simple, and developmentally appropriate way of assessing Criterion A Intimacy that sidesteps the limitations of self-report measures of intimacy, as “pictorial measures of this kind may bypass verbally encoded schemata that more strongly emphasize feeling close, and instead call forth a more deeply structured sense of self-other union” [(34), p. 610]. As the limitations of self-report measures may be especially pronounced for individuals with higher levels of personality pathology, the IOS is a promising tool for assessing aspects of personality pathology within this developmental stage.

## Data availability statement

The raw data supporting the conclusions of this article will be made available by the authors, without undue reservation.

## Ethics statement

The studies involving human participants were reviewed and approved by the Institutional Review Board of University of Houston. Written informed consent to participate in this study was provided by the participants’ legal guardian/next of kin.

## Author contributions

The study was conceived by CS who wrote the introduction and discussion. BC, SK, and SV contributed to data collection, data cleaning, data management, data analyses, and write up of the results and methods. All authors contributed to the article and approved the submitted version.

## Funding

The study was supported by funding from the McNair Family Foundation.

## Conflict of interest

The authors declare that the research was conducted in the absence of any commercial or financial relationships that could be construed as a potential conflict of interest.

## Publisher’s note

All claims expressed in this article are solely those of the authors and do not necessarily represent those of their affiliated organizations, or those of the publisher, the editors and the reviewers. Any product that may be evaluated in this article, or claim that may be made by its manufacturer, is not guaranteed or endorsed by the publisher.
